# The causal meaning of Hamilton’s rule

**DOI:** 10.1098/rsos.160037

**Published:** 2016-03-16

**Authors:** Samir Okasha, Johannes Martens

**Affiliations:** 1 Department of Philosophy, Cotham House, University of Bristol, Bristol BS6 6JL, UK; 2 Institute for the History and Philosophy of Science and Technology, University of Paris-Sorbonne, Paris, France

**Keywords:** Hamilton’s rule, altruism, causality, average effect

## Abstract

Hamilton’s original derivation of his rule for the spread of an altruistic gene (*rb*>*c*) assumed additivity of costs and benefits. Recently, it has been argued that an exact version of the rule holds under non-additive pay-offs, so long as the cost and benefit terms are suitably defined, as partial regression coefficients. However, critics have questioned both the biological significance and the causal meaning of the resulting rule. This paper examines the causal meaning of the generalized Hamilton’s rule in a simple model, by computing the effect of a hypothetical experiment to assess the cost of a social action and comparing it to the partial regression definition. The two do not agree. A possible way of salvaging the causal meaning of Hamilton’s rule is explored, by appeal to R. A. Fisher’s ‘average effect of a gene substitution’.

## 1. Introduction

Hamilton [1] derived his rule for the spread of an allele coding a social behaviour (*rb*>*c*) by assuming additivity of costs and benefits. This is a significant restriction as pay-off additivity is unlikely to be the rule in social interactions. There has been much discussion of how, if at all, Hamilton’s rule can be extended to cover non-additive pay-offs. One approach invokes weak selection to derive an approximate version of the rule [2–4]. (This is ‘*δ*-weak’ selection in the sense of Wild & Traulsen [5], i.e. phenotypically similar strategies.) Another approach argues that an exact version of the rule does in fact apply under pay-off non-additivity, so long as the cost and benefit terms are suitably defined, as partial regression coefficients [6–13]. This latter approach, dubbed the ‘regression method’ by Allen *et al.* [14], is the focus here.

The generalized version of Hamilton’s rule that results from the regression method is correct as a mathematical statement, but its biological significance is less clear. In Hamilton’s original work, the costs and benefits of a social action are described in explicitly causal terms, and the rule is meant to decompose natural selection into distinct causal components, as Frank [9] notes. However, Allen *et al.* [14,15] argue that the generalized Hamilton’s rule lacks causal meaning, so cannot yield insight into the causes of allele frequency change. Similarly, Birch & Okasha [16] query whether the *c* and *b* terms of the generalized Hamilton’s rule can be given a ‘causal interpretation’.

Our contribution here is twofold. Firstly, we provide an explicit measure of the causal effect of a social action on the actor’s pay-off, in the context of a simple model of social evolution, based on a hypothetical experimental intervention, and show that it does not correspond to the cost term of the generalized Hamilton’s rule. (The same applies to the benefit term.) Secondly, we outline an argument of Fisher [17] about how to measure the causal effect of a gene substitution in a non-additive genetic system. This argument has interesting connections with social evolution theory and suggests a way of salvaging the causal meaning of the generalized Hamilton’s rule.

## 2. Generalized Hamilton’s rule

To derive the generalized Hamilton’s rule, we begin with the Price equation [18] applied to a gene that codes for a social action. Assuming no mutation or gametic selection, the one generation change in the population-wide frequency of the gene is given by:

2.1 $$\Delta p=\frac{\text{Cov}(w_{i},p_{i})}{\bar{w}},$$

where *w*_*i*_ is the fitness (i.e. gametic output) of the *i*th individual, *p*_*i*_ is the frequency of the gene in the *i*th individual, *p* is the population-wide frequency of the gene and $\bar{w}$ is average population fitness. The covariance is taken over all individuals in the population.

**Figure 1.**
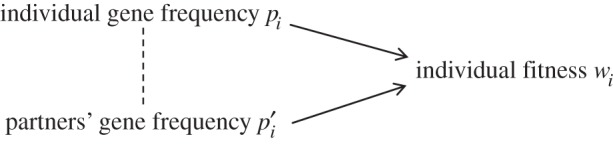
Direct and indirect determinants of fitness.

We assume that an individual’s fitness *w*_*i*_ depends on its own gene frequency *p*_*i*_ and the average gene frequency of its social partners *p*′_*i*_, which may be correlated (figure 1).

Following Queller [6], we then write *w*_*i*_ as a linear regression on *p*_*i*_ and *p*′_*i*_:

2.2 $$w_{i}=\alpha +\beta_{wp.p^{\prime }}p_{i}+\beta_{wp^{\prime }.p}p_{i}^{\prime }+e_{i},$$

where *α* is baseline fitness; *β*_*wp*.*p*′_ is the partial regression of an individual’s fitness on its own gene frequency, controlling for social partners’ gene frequency; *β*_*wp*′.*p*_ is the partial regression of an individual’s fitness on its social partners’ average gene frequency, controlling for individual gene frequency and *e*_*i*_ is the residual.

Equation (2.2) can then be substituted into equation (2.1), yielding

2.3 $$\Delta p=\frac{\left(\beta_{wp.p^{\prime }}+r\beta_{wp^{\prime }.p})\text{Var}(p\right)}{\bar{w}},$$

where *r*=*β*_*p*′*p*_=(*p*,*p*′)/(*p*) is the linear regression of social partners’ gene frequency on individual gene frequency, which is one standard definition of the coefficient of relatedness [4,6,8,9,11]. Equation (2.3) is a version of Hamilton’s rule in its ‘neighbour-modulated’ form, i.e. which considers the effect on a focal individual’s fitness of the genes (and hence actions) of its social partners.

As Hamilton [1] first showed, we can instead consider the effect of a focal individual’s genes (and hence actions) on the fitness of its social partners, rather than vice versa. The relevant regression coefficient corresponding to this effect is *β*_*w*′*p*.*p*′_, i.e. the partial regression of social partners’ fitness on an individual’s gene frequency, controlling for the social partners’ gene frequency. The coefficients *β*_*wp*′.*p*_ and *β*_*w*′*p*.*p*′_ are numerically identical [19], allowing equation (2.3) to be re-written as:

2.4 $$\Delta p=\frac{\left(\beta_{wp.p^{\prime }}+\beta_{w^{\prime }p.p^{\prime }}r)\text{Var}(p\right)}{\bar{w}}.$$

By labelling *β*_*wp*.*p*′_ and *β*_*w*′*p*.*p*′_ as ‘−*c*’ and ‘*b*’, respectively, (2.4) can be re-written as:

2.5 $$\Delta p=\frac{\left(rb-c)\text{Var}(p\right)}{\bar{w}},$$

which is a version of Hamilton’s rule widely found in the literature [6,10,12,13,20]. It tells us that the gene will spread so long as *rb*>*c*.

The generalized Hamilton’s rule of equation (2.5) makes minimal assumptions; it simply partitions the total evolutionary change into ‘direct’ and ‘indirect’ components, corresponding to the two pathways in figure 1. In particular, it can be applied whether the true relation between *w*, *p*_*i*_ and *p*′_*i*_ is linear or not; if it is nonlinear, then the *c* and *b* coefficients will be functions of population-wide gene frequency.

The generalized Hamilton’s rule raises interesting interpretive questions. Some have argued that the rule in this form has little explanatory value as it is simply a mathematical identity [14,15,21]; while others have seen the generality of the rule as an advantage, a proof that inclusive fitness theory does not rely on restrictive assumptions [10–12,22]. The issue of causality lies at the heart of this dispute.

## 3. Two-player game with synergy

To study the causal meaning of the generalized Hamilton’s rule, we follow Gardner *et al.* [12,20] in applying the rule to a well-known model of pairwise interaction with synergistic pay-offs [13,23,24]. An infinite population of haploid asexual organisms engage in pairwise social interactions in every generation. Organisms are of two types, altruists (A) and selfish (S). ‘A’ types perform an action that is costly for themselves but benefits their partner; ‘S’ types do not perform the action. The social action is assumed to affect only the actor and its partner, thus kin competition is assumed absent. Type is controlled genetically and perfectly inherited.

An organism’s pay-off from the social interaction depends on its own type and its partner’s type. Pay-offs are interpreted as increases in lifetime reproductive fitness over a unit baseline. Pay-offs to the actor are shown in table 1 above. Performing the action incurs a cost of *C* and confers a benefit of *B*, irrespective of partner type; if both actor and partner perform the action, each gets an additional synergistic benefit of *D*. So the parameter *D* quantifies the deviation from pay-off additivity when two A types are paired together. The natural interpretation of the model is that *C*>0 and *B*>0; but the analysis below assumes nothing about the sign of *B*, *C* or *D*.

**Table 1.** Two-player game with synergy.

		partner	
		A	S
actor	A	*B*−*C*+*D*	−*C*
	S	*B*	0

There are three pair-types in the population, AA, AS and SS, whose relative frequencies in the initial generation are *P*, 2*Q* and *R*, respectively, where *P*+2*Q*+*R*=1. The overall frequency of the A type is *p*=*P*+*Q*.

The pattern of assortment, i.e. pairing rule, is described by the coefficient of relatedness, *r*, defined as the regression of partner type on actor type. Let *p*_*i*_=1 if the *i*th organism is an A, *p*_*i*_=0 otherwise; and *p*′_*i*_=1 if the *i*th organism is paired with an A, *p*′_*i*_=0 otherwise. Then, *r* = *β*_*p*′*p*_=*Cov*(*p*′,*p*)/*Var*(*p*). An explicit expression for *r* can be written in terms of *P*, *Q* and *R* as:

3.1 $$r=\frac{P-{\left(P+Q\right)}^{2}}{\left(P+Q)(R+Q\right)}.$$

Conversely, we can express *P*, *Q* and *R* in terms of *r* and *p*, as shown in table 2.

**Table 2.** Pair-type frequencies.

pair-type	frequency
AA	*P*=*p*^2^+*rp*(1−*p*)
AS	2*Q*=2*p*(1−*p*)(1−*r*)
SS	*R*=(1−*p*)^2^+*rp*(1−*p*)

We can also write the conditional probability that an organism (or ‘actor’) has a partner of either type, given its own type, in terms of *r* and *p*, as shown in table 3 below.

**Table 3.** Conditional probabilities.

Pr(partner is A | actor is S)=p(1−r)
Pr(partner is S | actor is S)=1−p(1−r)
Pr(partner is A | actor is A)=r+p(1−r)
Pr(partner is S | actor is A)=(1−p)(1−r)

To apply the generalized Hamilton’s rule, we can write the *b* and *c* coefficients of equation (2.5) in terms of *r*, *p*, and the three pay-off parameters *B*, *C* and *D* (following Gardner *et al.* [20]). This yields

3.2 $$-c=-C+\frac{r+p(1-r)}{1+r}D$$

and

3.3 $$b=-B+\frac{r+p(1-r)}{1+r}D.$$

The generalized Hamilton’s rule then yields an expression for the one-generation evolutionary change

3.4 $$\Delta p=\{rB-C+D[r+p(1-r)]\}\frac{\mathrm{Var}(p)}{\bar{w}}.$$

Note that *Δp* is a function of *p*, so selection is frequency-dependent. Polymorphic equilibrium will obtain when *p*=[*C*−*r*(*B*+*D*)]/*D*(1−*r*); the stability of the equilibrium depends on the sign of *D*.

### 3.1 Causal analysis

To determine the causal effect of the social action, consider the following experimental intervention. We randomly pick an S type from the population and switch it to A (e.g. by mutation), while leaving everything else unchanged, and consider the effect on the actor’s pay-off. (A similar analysis applies to partner pay-off.) Doing an experimental intervention of this sort is the standard way to assess causality in science, and is often taken to define the causal relation [25]. In the context of social evolution models, such ‘switching’ has often been discussed as a way of assessing the cost of a social action [15,26–28].

If the chosen S type (the actor) has an A partner, then switching will increase the actor’s pay-off by (−*C*+*D*). If the actor has an S partner, then switching will increase the actor’s pay-off by −*C*. The expected effect of the S→A switch on the actor’s pay-off is, therefore:

*Expected causal effect of S→A switch on actor’s pay-off*

3.5 $$\begin{array}{rl} & =(-C+D)\cdot \mathrm{Pr}(\text{partner is A}\;|\;\text{actor is S})+(-C)\cdot \mathrm{Pr}(\text{partner is S}\;|\;\text{actor is S})\\ & =(-C+D)\cdot p(1-r)+(-C)\cdot [1-p(1-r)]\\ & =-C+D\cdot p(1-r). \end{array}$$

The quantity in equation (3.5) is called the expected *causal* effect because it describes the result of an experimental intervention, so is not simply a population statistic like a correlation or regression coefficient [25,29]. The experiment consists in switching a randomly chosen S type into an A, while holding fixed its partner, and considering the effect on the actor’s pay-off; its outcome is described by a random variable which can take two values, (−*C*+*D*) or (−*C*), with probabilities determined by the conditional probability that the actor has a partner of each type; the expected value of this random variable is equation (3.5).

Another way to interpret (3.5) is to consider a cohort of S types drawn from the population. Some members of the cohort will be partnered with an S, others with an A. If the cohort is representative of the population, the proportions partnered with an S and an A will be Pr(partner is A | actor is S) and Pr(partner is S | actor is S), respectively. Suppose that all members of the cohort then switch from S to A while their partners are held fixed. This will cause a *per capita* change in personal pay-off equal to the expected effect in equation (3.5).

Note that the expected causal effect of an S→A switch on the actor’s pay-off in (3.5) is not equal but opposite in sign to the expected causal effect of the reverse A→S switch (unless *D*=0 or *r*=0). This is because a randomly chosen A faces different probabilities of having a partner of each type than does a randomly chosen S.

Now compare equation (3.5) with the −*c* term in the generalized Hamilton’s rule (equation (3.2)). The −*c* term is also a weighted average of (−*C*+*D*) and (−*C*), but with different weights. Equations (3.5) and (3.2) are identical in exactly three cases: (i) *D*=0; (ii) *r*=0 and (iii) *p*=1/(1−*r*). Case (i) is where pay-offs are additive; case (ii) is where pairs are formed at random; case (iii) is only possible if *r*≤0, i.e. negative assortment, and for fixed *r* the equality in question will obtain for only one value of *p*. Thus with non-additive pay-offs, the −*c* term of Hamilton’s rule and the expected causal effect of an S→A switch on actor’s pay-off will almost always differ in magnitude, and may differ in sign.

It is useful to express −*c* in a form that permits direct comparison with the expected causal effect:

3.6 $$-c=(-C+D)\frac{\mathrm{Pr}(\text{partner is A}\;|\;\text{actor is A})}{k}+(-C)\frac{\mathrm{Pr}(\text{partner is S}\;|\;\text{actor is S})}{k},$$

k=[Pr(partner is A | actor is A)+Pr(partner is S | actor is S)].

In equation (3.6), the weights on (−*C*+*D*) and (−*C*) are proportional to Pr(partner is A | actor is A) and Pr(partner is S | actor is S), respectively. (Note that these two probabilities do not sum to one, hence the normalizing term *k* in the denominator.)

When −*c* is written this way, its oddity as a measure of the causal effect of the social action becomes apparent. The components in the sum, i.e. (−*C*+*D*) and (−*C*), have an obvious causal meaning; they are the changes to an actor’s personal pay-off caused by an S→A switch, depending on whether it is partnered with an A or an S. But the weights on these two changes do not equal the proportion of S types in the population with a partner of each sort; so −*c* is not the *per capita* change in personal pay-off that would result if a representative cohort of S types were switched to A.

The discrepancy between −*c* and the expected causal effect is due to non-additivity. In general, a partial regression coefficient only corresponds to the expected effect of an experimental intervention of the sort described here—in which one independent variable is increased by a unit while the other(s) are held fixed—if the linear model describes the true relation between the variables [29,30]. In the current case, where a linear model has been fitted to nonlinear data, −*c* does not equal the expected effect on actor’s pay-off resulting from experimentally switching a randomly chosen S type to A while holding its partner fixed.

This analysis supports the view that the −*c* term of the generalized Hamilton’s rule, while useful for describing evolutionary change, lacks a natural causal interpretation. Experimental determination of the cost of the social action, via the experiment described above, will not agree with the cost as measured by the partial regression coefficient. Parallel remarks apply to the relation between the *B* term of Hamilton’s rule and the expected effect of an S→A switch on partner pay-off.

## 4. Fisher to the rescue?

One way of salvaging the causal meaning of Hamilton’s rule is to draw on an argument made by Fisher [17] in a different context, recently revisited by Lee & Chow [31]. To derive his fundamental theorem of natural selection, Fisher [32] introduced a notion he called ‘the average effect of a gene substitution’ on a quantitative character of interest. This was intended as a measure of the effect, on average in a population, of a given gene being substituted for one of its alleles (e.g. by mutation), and was defined by Fisher as the linear regression of an individual’s character value on the number of copies of the gene in its genotype (=0,1 or 2 for diploids).

Fisher [17] focuses on the average effect of a gene substitution in a one-locus two-allele Mendelian model with dominance. There are three genotypes *A*_1_*A*_1_, *A*_1_*A*_2_ and *A*_2_*A*_2_ with frequencies of *P*, 2*Q* and *R*; character values are *w*_*A*_1_*A*_1__, *w*_*A*_1_*A*_2__ and *w*_*A*_2_*A*_2__ (table 4). Random mating is not assumed, so genotypes need not be in Hardy–Weinberg proportions. The effect of an $A_{1}\to A_{2}$ substitution depends on whether the substituted gene is in a homozygote or heterozygote, i.e. on whether the change is from $A_{1}A_{1}\to A_{1}A_{2}$ or from $A_{1}A_{2}\to A_{2}A_{2}$. The former substitution changes an individual’s character by [*w*_*A*_1_*A*_2__−*w*_*A*_1_*A*_1__], the latter by [*w*_*A*_2_*A*_2__−*w*_*A*_1_*A*_2__]. The average effect of the gene substitution is a weighted average of these two quantities.

**Table 4.** One-locus two-allele model.

genotype	frequency	character value
*A*_1_*A*_1_	*P*	*w*_*A*_1_*A*_1__
*A*_1_*A*_2_	2*Q*	*w*_*A*_1_*A*_2__
*A*_2_*A*_2_	*R*	*w*_*A*_2_*A*_2__

Although Fisher’s average effect is defined statistically, via the linear model, he also gives it a causal interpretation. Fisher [17] *appears* to claim that the average effect of an $A_{1}\to A_{2}$ substitution equals the average change in character if a randomly picked *A*_1_ allele were experimentally changed into an *A*_2_ while everything else is held constant. Intuitively this interpretation is suspect, given that a linear model has been fitted to a nonlinear system; and indeed Falconer [33] argued that Fisher’s interpretation was incorrect, by computing the expected effect of a hypothetical $A_{1}\to A_{2}$ mutation and showing that it does not, in general, equal Fisher’s average effect of a gene substitution. However, more recently Lee & Chow [31], building on Edwards [34], have unpacked Fisher’s curious logic and argued that, in a sense, the average effect can be imbued with a causal meaning even under dominance.

The issue is formally similar to our social evolution problem. Our three pair-types correspond to the three genotypes in Fisher’s model; personal pay-off corresponds to character value; and an S→A switch corresponds to an $A_{1}\to A_{2}$ substitution. In both cases, the effect of the switch (or substitution) is context-dependent, due to the nonlinearity; and in both cases we have a measure of the average effect of the switch (or substitution) defined by fitting a linear model. Thus, the −*c* term in the generalized Hamilton’s rule corresponds to Fisher’s average effect of a gene substitution.

The key point is that Fisher was concerned with the effect of a gene substitution ‘in the population as actually constituted’, as Lee & Chow [31] emphasize. Fisher understood the ‘constitution’ of the population to include the rules by which the genes are combined into genotypes, i.e. the mating pattern. Substituting a number of *A*_1_ genes by *A*_2_ genes might break the rules of combination in the population, i.e. require an implicit change in the mating pattern, so the actual effect of such an intervention might not correspond to the effect that Fisher was concerned with.

Fisher [17] introduces a particular measure λ of the deviation from random mating in the population, defined by λ=*Q*^2^/*PR*. With random mating, i.e. Hardy–Weinberg proportions, λ=1; with assortative mating λ>1. (Importantly, λ is not the only possible measure of deviation from random mating; see §(b).) Constancy of the mating pattern, in Fisher’s discussion, means constancy of λ.

Now consider the effect of an $A_{1}\to A_{2}$ substitution on λ. If the substitution is of the $A_{1}A_{1}\to A_{1}A_{2}$ sort then it will reduce λ, while if it is of the $A_{1}A_{2}\to A_{2}A_{2}$ sort then it will increase λ. Suppose we pick a cohort of *A*_1_ genes and substitute them with *A*_2_ genes. In order for this intervention to leave λ unchanged, the *A*_1_ genes in the cohort must come from *A*_1_*A*_1_ and *A*_1_*A*_2_ individuals in specific proportions; so the cohort must be carefully chosen. What Fisher [17] shows is that the *per capita* change in character value, in the cohort, then equals the average effect of the gene substitution as defined by the linear model.

An equivalent way to formulate Fisher’s result is this. The average effect of an $A_{1}\to A_{2}$ substitution, as defined by the linear model, is a weighted average of [*w*_*A*_1_*A*_2__−*w*_*A*_1_*A*_1__] and [*w*_*A*_2_*A*_2__−*w*_*A*_1_*A*_2__], which are the changes in individual character value that result from an $A_{1}A_{1}\to A_{1}A_{2}$ and an $A_{1}A_{2}\to A_{2}A_{2}$ mutation, respectively. The weights are defined by the proportions of *A*_1_*A*_1_ and *A*_1_*A*_2_ individuals in a cohort which is such that, when all the *A*_1_ genes in the cohort are switched to *A*_2_, λ is unchanged. Importantly, the proportions of *A*_1_*A*_1_ and *A*_1_*A*_2_ individuals in such a cohort will in general not equal their population-wide proportions.

This goes some way towards reconciling Fisher’s statistical definition of the average effect with the hypothetical experimental intervention he describes. Falconer [33] was right that Fisher’s average effect is not equal to the expected character change of an $A_{1}\to A_{2}$ substitution if the *A*_1_ gene is picked at random from the whole population. However, if the *A*_1_ gene is picked at random from a cohort which meets Fisher’s ‘constant λ’ condition, then the resulting expected change is equal to the average effect of the gene substitution as defined by Fisher.

The precise significance of this in the context of Fisher’s own discussion is a delicate matter. However, our interest here is in applying Fisher’s argument to our social evolution problem.

### 4.1 Application to social evolution

Returning to the pairwise interaction model, consider the following experiment. We pick any cohort of S types from the population and switch them to A. As a result of this intervention, *P*, *Q* and *R* increase by *P*, *Q* and *R*, respectively, where *P*+*Q*+*R*=0; therefore *p* increases by *p*=*P*+*Q*.

When an S in an AS pair is switched to A, its pay-off increases by (−*C*+*D*); when an S in an SS pair is switched to A, its pay-off increases by (−*C*). The ratio of the two types of switches is *P*:−*R*. Therefore, the *per capita* change in actor’s pay-off caused by the experimental intervention equals:

4.1 $$\frac{dP(-C+D)-dR(-C)}{dP-dR}.$$

The above expression holds true irrespective of how the cohort of S types is chosen. If the cohort is chosen at random, i.e. contains S types with A and S partners in identical proportions to those in the global population of S types, then *P*:−*R*=*Q*:*R*, and (4.1) is then equal to the expected causal effect of an S→A switch on the actor’s pay-off, as defined by equation (3.5).

Now consider the −*c* term in Hamilton’s rule, given in equation (3.2). Following Fisher’s logic, we equate −*c* with the *per capita* change in actor’s pay-off caused by the experimental intervention (4.1), and extract a constraint on *P*, *Q* and *R*. Equating (3.2) and (4.1) gives:

$$\frac{dP(-C+D)-dR(-C)}{dP-dR}=-C+\frac{r+p(1-r)}{1+r}D.$$

We then make the following substitutions: −*R*=*P*+2 *Q*; *p*=*P*+*Q*; *P*=*rp*+*p*^2^(1−*r*) and *r*=[*P*−(*P*+*Q*)^2^]/[(*P*+*Q*)(*R*+*Q*)]. After simplifying, this gives

dP(QR−QP)=2 dQ(PR+PQ).

Dividing across by *PQR* and further simplifying yields

$$\left(\frac{\text{d}P}{P}\right)+\left(\frac{\text{d}R}{R}\right)=2\left(\frac{\text{d}Q}{Q}\right).$$

Taking the infinite limit, i.e. letting *P*, *Q* and *R* become arbitrarily small, then integrating both sides and combining the constants of integration

4.2 $$\frac{Q^{2}}{PR}=\text{const.}=\lambda .$$

This is precisely Fisher’s ‘constant λ’ condition and shows the close link between our social evolution problem and his population-genetic problem. The meaning of equation (4.2) is worth rehearsing. A cohort of S types, some in AS and some in SS pairs, was chosen from the population and experimentally switched to A. We then asked the question: under what condition is the *per capita* change in actor’s pay-off that results from the experimental intervention equal to −*c*? The answer is given by equation (4.2): the cohort must be chosen in such a way that the intervention leaves the ratio *Q*^2^/*PR* unchanged. This then determines the proportions of S types in the cohort with A and S partners, respectively.

It follows that the −*c* term of Hamilton’s rule does have a quasi-causal meaning, even with non-additive pay-offs. As we know, −*c* is not the expected change in actor’s pay-off if a randomly chosen S type is switched to A. However, if the S to be switched is chosen not at random from the population, but rather at random from any cohort of S types satisfying the ‘constant λ’ condition, then the resulting expected change in pay-off is equal to −*c*. If we regard the assortment pattern as an ‘environmental’ parameter, quantified by λ, and wish to measure the causal effect of an S→A switch on actor’s pay-off in a constant environment, then −*c* is arguably the correct measure. A parallel analysis applies to the *b* term.

### 4.2 Constant *r* versus constant λ

This Fisherian defence of the causal meaning of the generalized Hamilton’s rule rests on three premises: first, that in assessing the causal effect of an S→A switch the environment should be held constant; second, that the assortment pattern is part of the environment; and third, that λ is the appropriate measure of assortment.

The third premise is the hardest to defend. For an alternative measure of assortment is simply *r* itself, the coefficient of relatedness between social partners. Recall the respective definitions of *r* and λ in terms of *P*, *Q* and *R*:

$$\begin{array}{rl} r & =\frac{P-{\left(P+Q\right)}^{2}}{\left(P+Q)(R+Q\right)}\\ \mathrm{and}\;\lambda & =\frac{Q^{2}}{PR}. \end{array}$$

Algebraic manipulation shows that *r* and λ are related as follows:

4.3 $$\lambda =\frac{Q(1-r)}{Q(1-r)+r}.$$

Equation (4.3) shows that *r* and λ both quantify the deviation from random assortment. Note that *r* ranges from −1 to +1, while λ ranges from 0 to +∞. With random assortment, *r*=0 and λ=1; with perfect assortment, *r*=1 and λ=0. However, constancy of λ across generations does not imply constancy of *r*, nor vice versa. The numerical example below illustrates this point.

Example

In generation 1, $p=\frac{1}{2}$, $r=\frac{1}{2}$.

Therefore, $P=\frac{3}{8}$, $2Q=\frac{1}{4}$, $R=\frac{3}{8}$ and $\lambda =\frac{1}{9}$.

Evolution occurs, leading *p* to increase to $\frac{3}{4}$.

Case (i): *r* stays constant

So in generation 2, $p=\frac{3}{4}$, $r=\frac{1}{2}$.

Therefore, $P=\frac{21}{32}$, $2Q=\frac{6}{32}$, $R=\frac{5}{32}$ and $\lambda =\frac{3}{35}.$

So *r* has stayed constant while λ has decreased.

Case (ii): λ stays constant

So in generation 2, $p=\frac{3}{4}$, $\lambda =\frac{1}{9}$.

Therefore, *P*≈0.65, 2*Q*≈0.20, *R*≈0.15.

This gives *r*≈0.47. So λ has stayed constant while *r* has decreased.

Fisher [17] offers no independent argument for why λ is the ‘correct’ measure of deviation from random mating in his population-genetic model, nor therefore for why environmental constancy should mean constancy of λ. Rather, he simply shows that constancy of λ is implied if we equate the average effect of a gene substitution, as defined by the linear model, with the *per capita* effect caused by an experimental gene substitution.

The same applies to our social evolution model. If environmental constancy were defined as constancy of *r* rather than λ, this would imply different weights on the two sorts of S→A switch; and if we computed the expected effect of an S→A switch on actor’s pay-off using these weights, the result would not equal −*c*. In the absence of an independent reason to hold λ rather than *r* fixed, the Fisherian defence of the causal meaning of Hamilton’s rule, above, cannot be considered logically watertight.

## 5. Conclusion

With additive pay-offs, the −*c* and *b* terms of Hamilton’s rule have a clear causal meaning: they equal the amount by which an actor would increase its own and its social partner’s pay-off, respectively, by performing the social action, i.e. switching from S to A. With non-additivity the situation is different, as the effect of the switch depends on context, i.e. partner type. As the expected effect of an S→A switch on actor’s pay-off does not equal the −*c* term, and similarly for *b*, the causal meaning of the generalized Hamilton’s rule, derived from the regression method, is called into question.

A possible way of rescuing the causal meaning of the −*c* and *b* terms involves adapting Fisher’s idea that his ‘average effect of a gene substitution’, in a non-additive genetic system, corresponds to the expected result of an experimental intervention in a ‘constant environment’, so does have a causal meaning. An analogous argument applies exactly to our social evolution model with synergistic pay-offs. However, like Fisher’s original, the argument has an intrinsic limitation in that it relies on a particular way of defining ‘environmental constancy’ that lacks independent justification.

The upshot, therefore, is that the defenders and critics of the generalized Hamilton’s rule are both partly right. When −*c* and *b* are defined via the regression method, they do not correspond to the cost and benefit of the social action as measured by a standard experimental determination. However, it does not follow that Hamilton’s rule is devoid of all causal meaning. For as Fisher shows, his average effect can be interpreted as the expected outcome of an experimental intervention of a very particular sort, and precisely the same is true of the components of Hamilton’s rule.

Finally, note that while the generalized Hamilton’s rule was derived with minimal assumptions, our analysis of its causal meaning, and the connection to Fisher’s argument, was done in the context of the rule as applied to a simple evolutionary model. Do the morals we have drawn apply to more complex models, e.g. that incorporate population structure, kin competition and multiple partners?

Our negative result—that the −*c* term does not equal the expected outcome of an experiment in which a randomly chosen S is switched to A—will apply wherever *c* is frequency-dependent, i.e. wherever the social action has non-additive effects on fitness. Our positive result—that −*c* does equal the expected outcome of an S→A switch in a ‘constant environment’—relies on a definition of environmental constancy that is specific to the pairwise interaction model examined here. It seems likely that a similar result will hold for more complex models, given a suitable definition of environmental constancy; however, this needs to be examined on a case-by-case basis.
